# Effects of imagery training on cognitive function among collegiate athletes: a randomized controlled trial

**DOI:** 10.3389/fcogn.2026.1826442

**Published:** 2026-08-10

**Authors:** Yingke Li, Nurwina Anuar, Simon B. Cooper

**Affiliations:** 1 Faculty of Education, Universiti Kebangsaan Malaysia, Bangi, Selangor, Malaysia; 2 Department of Sport Science, Sport Health and Performance Enhancement (SHAPE) Research Centre, Exercise and Health Research Group, Nothingham Trent University, Nottingham, United Kingdom

**Keywords:** cognitive function, imagery, executive function, memory, attention, collegiate athletes, squat

## Abstract

Cognitive functions are essential for athletic performance but are often compromised by fatigue, stress, and the combined demands of sport and academic study. Both physical exercise and motor imagery shown to influence cognition, but, their comparative acute effects remain underexplored in collegiate athletes. This study compared the effects of squat imagery, squat execution, and quiet rest on cognitive performance. Ninety-eight collegiate athletes were randomly assigned to a squat imagery group (30-min seated squat imagery; *n* = 36), a squat execution group (five sets of 1-min squats separated by 10-s rests; *n* = 32), or a control group (30-min seated rest; *n* = 30). Cognitive performance was assessed before and after the interventions using the Stroop, Flanker, Sternberg, and Visual Search tasks. Two-way repeated-measures analysis of variance (group × time) was conducted, and the Benjamini–Hochberg false discovery rate (FDR) procedure was applied to 18 primary interaction tests. Both squat imagery and execution improved cognitive performance, particularly inhibitory control and working memory. Significant group × time interactions were found for Stroop response time (simple and complex) and Sternberg response time (one- and three-item conditions; all *p* < 0.01, partial η^2^ = 0.10–0.14). Follow-up analyses showed these improvements were primarily driven by the squat imagery group. Compared with squat execution, squat imagery produced greater reductions in response time for the Stroop simple and Sternberg three-item conditions, while improvements in the Stroop complex and Sternberg one-item conditions were comparable. Squat imagery also produced greater improvements in Visual Search congruent response time than both squat execution and control. A significant group × time interaction was observed for Visual Search incongruent accuracy (*p* < 0.05, partial η^2^ = 0.08–0.14), reflecting reduced accuracy following squat execution, whereas performance remained stable in the squat imagery and control groups. Although Flanker incongruent accuracy initially showed a significant interaction (*p* = 0.032, partial η^2^ = 0.07), this effect did not survive FDR correction (*p*FDR = 0.082). These findings demonstrate that acute squat imaging enhances inhibitory control and working memory without the drawbacks of physical activity. Squat visualization serves as a low-intensity, pragmatic cognitive improvement for collegiate athletes.

## 1 Introduction

In competitive sports, optimal performance relies not only on physical capacity but also on the ability to adapt to rapidly changing situations and make effective decisions under pressure (Appelbaum and Erickson, 2018; Ryu et al., 2013). This capacity, supported by cognitive function, is fundamental for interpreting complex game scenarios, anticipating opponents' actions, and executing timely responses (Yongtawee et al., 2021). In fast-paced team sports, athletes must often process large volumes of information under strict time constraints and translate this information into accurate motor responses in real time (Alves et al., 2013; Gilbert et al., 2023). Cognitive performance is therefore an important contributor to sporting success and long-term athletic development (Kalén et al., 2021; Yongtawee et al., 2021).

Cognitive function refers to the essential capacities for perceiving, processing, storing, and applying information from both internal and external sources (Schmitt et al., 2006). It encompasses multiple domains, including executive function, memory, attention, perception, language, and psychomotor skills, each of which supports essential aspects of daily behavior and decision-making (Schmitt et al., 2006). These functions are fundamental to learning, problem-solving, and purposeful action, making cognitive function a central element in human performance across academic, occupational, and athletic contexts (Logan et al., 2023).

Collegiate athletes face a unique set of challenges that distinguish them from both professional athletes and non-athlete students. In addition to the demands of high-intensity training and competitive performance, they must simultaneously manage academic responsibilities and psychosocial pressures (Harper and Quaye, 2009; Rutledge II, 2023). Managing these dual demands necessitates sustained cognitive effort, encompassing resilience, effective decision-making, and self-regulation in addition to physical preparedness (Simpson et al., 2021; Smith et al., 2021). Consequently, strategies that may support cognitive performance without adding substantial physical load could be particularly valuable for collegiate athletes, with motor imagery representing one potentially useful approach.

Motor imagery is a psychological technique in which an individual mentally simulates an action without physically executing it (Murphy and Martin, 2002). It involves the intentional generation of sensory representations related to movement, including visual and kinesthetic characteristics (Marks, 2019; Morris et al., 2005). Research shows that by constructing sports-related images, individuals can mentally simulate the movement process and action essentials. This simulation process may support movement preparation and skill learning and has also been associated with selected physical and performance-related outcomes, including muscular strength and endurance performance (McCormick et al., 2015; Tod et al., 2015). It also contributes to the development of psychological skills essential for competitive success (Mamassis and Doganis, 2004; Sheard and Golby, 2006).

Evidence from both clinical and healthy populations suggests that structured imagery interventions may influence cognitive outcomes, including working memory, attention, and executive function (Mahmoud and Hafez, 2018; Srisupornkornkoola et al., 2023; Tien and Chang, 2024). Neurophysiological and neuroimaging studies have also reported changes in neural activity during or following imagery, including altered alpha-band activity and engagement of frontal and parietal regions involved in attention, working memory, and cognitive control (Johnson et al., 2019; Zemla et al., 2023; Moriya and Sakatani, 2017; Papadelis et al., 2007; Srisupornkornkoola et al., 2023). However, these findings should not be interpreted as evidence that all imagery interventions produce identical cognitive or neural effects, particularly when imagery content, duration, population, and outcome measures differ.

One theoretical explanation for the effects of motor imagery is functional equivalence, which proposes that imagined and physically executed actions share some cognitive and neural processes, although the two experiences are not identical (Kosslyn, 1996). Through the internal simulation of movement, individuals may rehearse task-relevant representations without overt physical execution (Holmes and Collins, 2001). However, despite growing evidence for the cognitive benefits of imagery in clinical and athletic populations, its effects in collegiate athletes remain underexplored. In particular, few studies have systematically examined sport-specific imagery as a means of enhancing cognitive function in this population.

Squat exercise was selected because it is a fundamental lower-body movement that is familiar to athletes across a wide range of sporting backgrounds. It is also relatively simple to standardize and can be performed or imagined without requiring sport-specific equipment or highly specialized technical skills. Previous studies have indicated that acute squat exercise may influence executive function and inhibitory control (Horiuchi et al., 2023; Tsukamoto et al., 2017), Based on functional equivalence, imagery of the same movement may engage some of the motor and cognitive processes involved in physical execution (Glover et al., 2020; Papadelis et al., 2007). Nevertheless, it remains unclear whether a single session of squat imagery produces cognitive effects comparable to those of squat execution, particularly among collegiate athletes who must manage both sporting and academic demands.

Therefore, the aim of the present study was to investigate the acute effects of squat imagery and squat execution on cognitive performance in collegiate athletes using a three-group randomized controlled design. Participants were assigned to a squat imagery group, squat execution group, or quiet-rest control group. Cognitive performance was assessed across inhibitory control, working memory, attentional control, and visual attention using the Stroop, Sternberg, Flanker, and Visual Search tasks. It was hypothesized that both squat imagery and squat execution would produce greater improvements in cognitive performance than quiet rest. It was further hypothesized that the effects of squat imagery would be comparable to, or greater than, those of squat execution.

## 2 Materials and methods

### 2.1 Participants characteristics

An *a priori* power calculation was performed using G^*^Power version 3.1.9.6 (Heinrich Heine University Düsseldorf, Düsseldorf, Germany) to estimate the required sample size. The calculation was based on a conservative medium effect size estimate (*f* = 0.20), an alpha level of 0.05, and statistical power of 0.90, following previous recommendations (Cohen, 2013; Kang, 2021). Based on these parameters, the analysis indicated that a total sample size of 84 was required to detect such an effect. Our recruited sample of 98 athletes exceeded this requirement, with participants recruited from the National University of Malaysia. Participants were randomly assigned to one of three groups: the squat imagery group (*n* = 36), squat execution group (*n* = 32), or control group (*n* = 30). The overall sample comprised 65 male and 33 female athletes with a mean age of 22.41 years (SD = 0.99). The overall demographic and sport-related characteristics of the participants are presented in Table 1.

**Table 1:** Characteristics of the participants.

Variable	Category	*N*	%
Age (M ± SD)		22.41 ± 0.99	
Gender	Male	65	66.3
	Female	33	33.7
Ethnicity	Malay	88	89.8
	Chinese	4	4.1
	Others	6	6.1
Religion	Islam	92	93.9
	Christian	4	4.1
	Buddhist	2	2.0
Sports type	Individual	41	41.8
	Team	57	58.2
Play years	< 7 years	43	43.9
	≥7 years	55	56.1
Competitive level	Expert	23	23.5
	Proficient	24	24.5
	Competent	30	30.6
	Advanced beginner	16	16.3
	Recreational	5	5.1
Achievement level	Elite/professional	32	32.7
	Competent	29	29.6
	Novice	37	37.8
Imagery use	Always	59	60.2
	Sometimes	38	38.8
	Never	1	1.0

### 2.2 Study design

The study was approved by the Institutional Ethics Committee of the University Kebangsaan Malaysia with the code of JEP-2024-1090, and all procedures complied with the ethical standards of the 1964 Helsinki declaration and its later amendments. Written informed consent was obtained from all participants prior to their involvement in the study.

A three-arm randomized controlled design was employed. Participants were randomly allocated to the squat imagery group, squat execution group, or control group using a computer-generated random sequence. All participants attended a familiarization session 7 days before the experimental session. During this session, participants were introduced to the computerized cognitive tasks, including the Stroop task, Sternberg paradigm, Flanker task, and Visual Search task. Standardized instructions were provided, and participants completed practice trials for each task to ensure task comprehension and procedural familiarity. No data from the familiarization session were included in the analyses (Moriya and Sakatani, 2017; Zemla et al., 2023).

### 2.3 Protocol

Standardized testing procedures were maintained for all assessments to minimize potential distractions. Ambient noise was reduced by using noise-canceling headphones, and lighting was adjusted to maintain a consistent testing atmosphere. The cognitive function tests consisted of the Stroop test to assess inhibitory control and selective attention, the Sternberg paradigm to evaluate working memory, the Flanker task to measure attentional control and executive function, and the Visual Search test to index selective attention and visual perception (Miyake et al., 2000; Sternberg, 1969; Stroop, 1935).

Before each task, participants received written and verbal instructions, followed by short practice trials with accuracy feedback to ensure comprehension. Practice data were excluded from analyses. The tasks lasted approximately 17 min in total, and the primary outcome measures were the response time of correct responses (ms) and accuracy (%). The four cognitive tasks were administered in a fixed order: Stroop task, Sternberg paradigm, Flanker task, and Visual Search task. This fixed order was used to ensure that any potential order or fatigue effects were kept consistent across repeated administrations of the cognitive testing battery and across the three experimental groups. The same task paradigms and testing procedures were used at both pre-test and post-test; however, stimuli were randomly generated or randomly presented at each administration according to the programmed task parameters.

During the experimental session, participants first completed the pre-test cognitive assessment, followed by their assigned condition, and then completed the post-test cognitive assessment.

The squat imagery group completed a 30-min standardized guided imagery session while seated in a quiet room. The session was delivered using standardized verbal instructions provided by a trained researcher. Participants were instructed to imagine performing the squat movement from a first-person perspective, as if they were carrying out the movement themselves, without producing any overt physical movement. The imagery instructions incorporated both visual and kinesthetic components. For the visual component, participants were guided to imagine the body position and movement sequence involved in preparing for the squat, lowering the body, holding the squat position, and returning to the starting position. For the kinesthetic component, participants were asked to imagine the bodily sensations associated with the movement, including muscle tension, weight distribution, joint position, balance, and pressure through the feet. Throughout the session, participants were guided to mentally repeat the squat movement sequence and maintain attention on the imagined movement. The session was supervised by the researcher to ensure that participants remained engaged with the imagery task and did not physically execute the squat movement.

**Figure 1:**
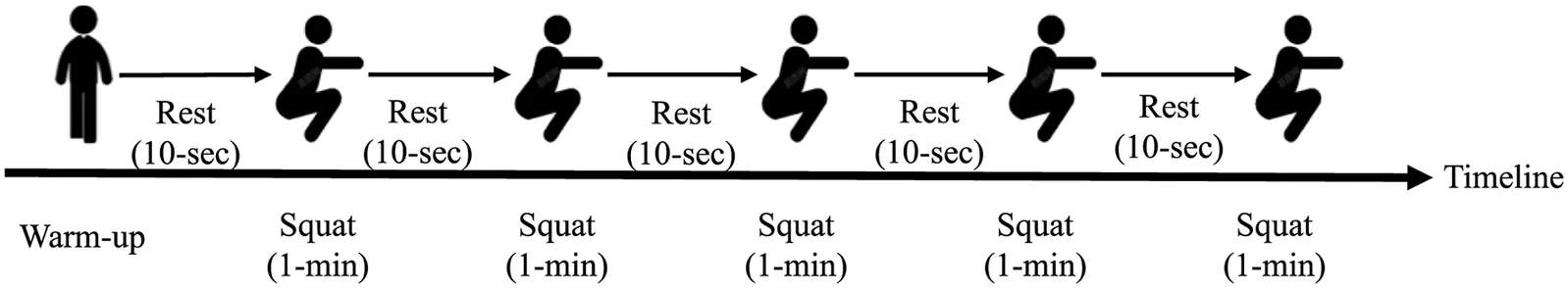
Overview of the squat execution protocol and assessment time points.

The squat execution group completed a standardized warm-up consisting of lunge holds, knee mobilization, and ankle mobility exercises, followed by five sets of 1-min static squats separated by 10-s inter-set rest intervals. The overall duration of the squat execution condition was approximately 30 min, including standardized instructions, warm-up, squat exercise, rest intervals, and transition time before the post-test assessment (see Figure 1).

The control group remained seated quietly for 30 min without receiving an active intervention.

**Figure 2:**
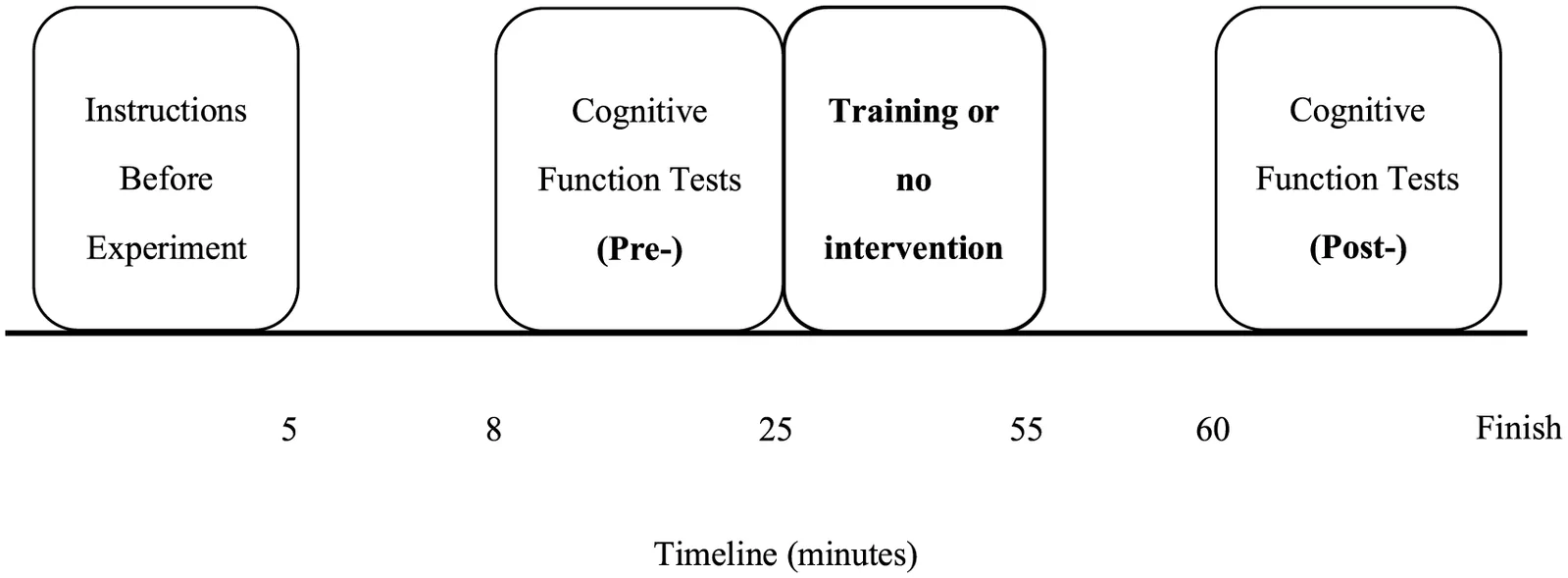
Visual representation of the experimental timeline.

The interval from the end of the pre-test assessment to the start of the post-test assessment was kept approximately comparable across the three groups. Post-test assessments commenced immediately after completion of each group's respective condition. Figure 2 provides schematic overviews of the study timeline.

### 2.4 Measures

#### 2.4.1 Stroop task

The Stroop test consists of two levels: simple and complex, assessing selective attention and executive function (specifically the domain of inhibitory control), respectively (Miyake et al., 2000). On both levels, a test word was presented at the center of the laptop screen, with a target and a distractor word randomly displayed on the left and right sides of the screen. Participants responded by pressing the appropriate arrow key (left or right) to indicate their choice.

On the simple level, the test word, target word, and distractor word were all presented in white font. The participant had to select the word which matched the target word in the center of the screen; a total of 20 stimuli were shown. On the complex level, the font color of the words did not match the semantic meaning of the word (e.g., the word “green” displayed in blue font), and participants were instructed to select the color in which the word was printed rather than the word itself. A total of 40 stimuli were presented in this level. Stimuli remained on the screen until the participant responded, with a 1-s inter-stimulus interval between trials.

#### 2.4.2 Sternberg test

The Sternberg Paradigm is a widely used test to measure the domain of working memory (Sternberg, 1969). The test comprises three levels with increasing complexity, each requiring a different working memory load (one-, three- and five-item). In the one-item level, participants identify the number “3” among 16 test stimuli. In the three-item and five-item levels, participants identify the target three (e.g., “A C H”) or five (e.g., “B F H M K”) randomly generated letters among 32 test stimuli, respectively. At the beginning of each level, the target items were presented with instructions to press the right arrow key for the target unit and the left arrow key otherwise. At every level, a selection stimulus was presented in the center of the screen, with a 1-s interval between stimuli.

#### 2.4.3 Flanker test

The Flanker task assesses attention control and executive function, consisting of two levels: congruent and incongruent (Eriksen and Eriksen, 1974). During the task, participants responded to stimuli presented on a computer monitor, which consisted of white arrows (< and >) on a black background, using arrow keys on the keyboard. Each trial presents five arrows, with a congruent stimulus showing all arrows facing the same direction (e.g., >>>>>, < < < < < ), whilst incongruent stimuli presented the central arrow in a different direction to the other arrows (e.g., < < > < <, >> < >>). Participants were instructed to press the arrow (left or right) key corresponding to the direction of the central arrow. The program displayed a total of 40 stimuli, including 10 sets of left arrows and 10 sets of right arrows for each condition (congruent and incongruent), with a 700-ms inter-stimulus interval.

#### 2.4.4 Visual Search test

The visual search test consisted of simple and complex levels, designed to assess selective attention and visual perception. In both levels, participants were instructed to press the space bar as soon as a target triangle was detected on the screen. After each response, a random delay (minimum 500-ms) occurred before a new target appeared.

On the congruent level, participants completed 20 trials in which the target was a triangle drawn with solid green lines on a black background. This level primarily assessed simple visuomotor speed. On the incongruent level, participants completed 40 trials. Here, background distractors were introduced, consisting of randomly moving dots that created a flashing visual effect. A new set of distractor dots was redrawn every 250-ms. The target triangle in this level was initially displayed with only a few visible points along each line, and the density of these points increased linearly over time until the participant responded (Gilbert et al., 2023).

### 2.5 Data analyses

Statistical analyses of the data were conducted using IBM SPSS Statistics 29.0 (IBM SPSS Inc., Chicago, IL, USA). Two-way mixed analyses of variance (ANOVAs), with group as the between-subjects factor and time as the within-subjects factor, were conducted for each cognitive task level and outcome variable. The between-subjects factor included three levels: squat imagery, squat execution, and control. The within-subjects factor included two levels: pre-test and post-test.

Partial eta squared (ηp^2^) was reported as the effect size for the main and interaction effects. To address the risk of inflated Type I error arising from the multiple analyses across four cognitive tasks, task levels, and two outcome measures, the Benjamini–Hochberg false discovery rate (FDR) procedure was applied to the 18 primary group × time interaction *p*-values. FDR-adjusted *p*-values were evaluated at the 0.05 level. Original and FDR-adjusted *p*-values are reported for interactions that were statistically significant before correction.

Group × time interactions that remained significant after FDR correction were examined using Bonferroni-adjusted simple-effects analyses to evaluate pre-to-post changes within each group. Change scores were calculated so that positive values represented improvement: pre-test minus post-test for response time and post-test minus pre-test for accuracy. Homogeneity of variance for the change scores was assessed using Levene's test. When the homogeneity assumption was satisfied, one-way ANOVA with Bonferroni-adjusted pairwise comparisons was used. When the assumption was violated, Welch's ANOVA and Games–Howell pairwise comparisons were applied. Analyses were conducted using available valid data; therefore, the degrees of freedom varied slightly across cognitive outcomes.

## 3 Results

Descriptive statistics (mean ± SEM) for each cognitive test are presented in Table 2. The Benjamini–Hochberg FDR correction was applied to the 18 primary group × time interaction tests. After FDR correction, six interactions remained significant: Stroop simple response time, Stroop complex response time, Sternberg one-item response time, Sternberg three-item response time, Visual Search congruent response time, and Visual Search incongruent accuracy. The group × time interaction for Flanker incongruent accuracy was significant before correction but did not remain significant after FDR correction.

**Table 2:** Cognitive function data across the imagery, execution and control groups.

Test	Test Level	Variable	Squat imagery		Squat execution		Control group	
			Pre	Post	Pre	Post	Pre	Post
Stroop	Simple	Response time [ms]	886 ± 30	783 ± 30	806 ± 33	805 ± 33	849 ± 33	852 ± 33
		Accuracy [%]	99.0 ± 0.5	98.5 ± 1.0	98.4 ± 0.5	97.7 ± 1.1	99.5 ± 0.5	96.8 ± 1.1
	Complex	Response time [ms]	1,207 ± 44	1,042 ± 41	1,153 ± 49	1,040 ± 45	1,173 ± 49	1,179 ± 45
		Accuracy [%]	97.5 ± 0.5	96.5 ± 0.8	97.8 ± 0.6	97.1 ± 0.9	97.4 ± 0.6	96.2 ± 0.9
Sternberg	One-item	Response time [ms]	562 ± 21	477 ± 21	500 ± 22	476 ± 23	547 ± 23	574 ± 23
		Accuracy [%]	98.4 ± 0.5	99.5 ± 0.8	98.7 ± 0.6	97.5 ± 0.9	98.1 ± 0.6	97.7 ± 0.9
	Three-item	Response time [ms]	654 ± 23	568 ± 20	611 ± 24	589 ± 22	656 ± 25	684 ± 22
		Accuracy [%]	98.9 ± 0.8	97.7 ± 0.6	99.0 ± 0.9	97.7 ± 0.6	96.0 ± 0.9	96.6 ± 0.6
	Five-item	Response time [ms]	804 ± 34	719 ± 28	764 ± 36	687 ± 29	776 ± 38	781 ± 30
		Accuracy [%]	97.1 ± 1.1	96.6 ± 1.0	97.6 ± 1.2	95.0 ± 1.1	93.4 ± 1.2	94.2 ± 1.2
Visual Search	Congruent	Response time [ms]	606 ± 17	549 ± 16	564 ± 18	567 ± 17	597 ± 19	617 ± 17
		Accuracy [%]	98.9 ± 1.9	95.4 ± 2.9	95.7 ± 1.0	97.5 ± 2.9	97.3 ± 1.4	94.8 ± 2.0
	Incongruent	Response time [ms]	1,175 ± 51	1,109 ± 48	1,095 ± 54	1,025 ± 51	1,311 ± 55	1,311 ± 52
		Accuracy [%]	95.7 ± 2.0	96.3 ± 2.1	95.8 ± 2.1	89.3 ± 2.2	95.8 ± 2.2	97.3 ± 2.3
Flanker	Congruent	Response time [ms]	541 ± 18	484 ± 28	535 ± 18	510 ± 28	607 ± 18	578 ± 28
		Accuracy [%]	99.3 ± 1.1	98.9 ± 2.5	98.2 ± 1.5	98.5 ± 2.6	97.4 ± 1.3	97.6 ± 2.4
	Incongruent	Response time [ms]	638 ± 39	557 ± 30	607 ± 43	560 ± 33	847 ± 44	733 ± 33
		Accuracy [%]	95.2 ± 4.0	92.7 ± 3.5	89.1 ± 4.2	89.6 ± 3.8	84.4 ± 4.3	90.7 ± 3.9

Data are mean ± SEM.

### 3.1 Stroop test

#### 3.1.1 Response time

**Figure 3:**
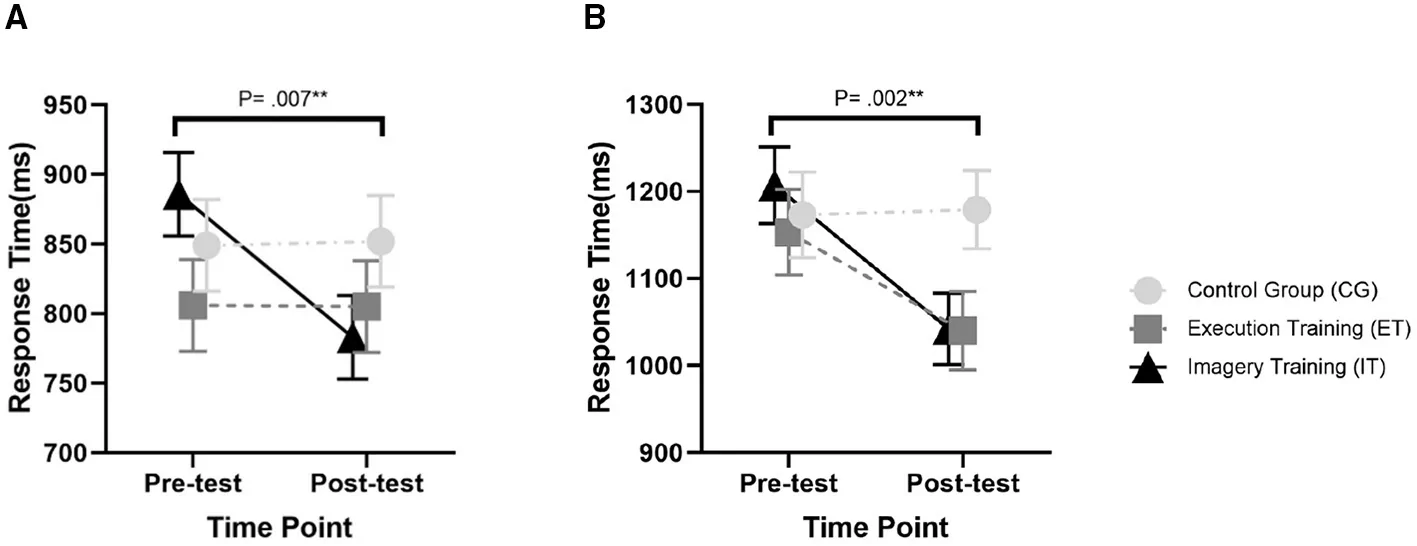
Response time (±SEM) for each group across pre- and post-test sessions in the Stroop task. **(A)** Shows the simple condition, and **(B)** shows the complex condition. IT, squat imagery group; ET, squat execution group; CG, control group. The *p*-values shown in the figure indicate the original group × time interaction effects; FDR-adjusted *p*-values are reported in the text. A significant group × time interaction was observed (*P* = .007; *P* = .002).

Response times on the simple level of the Stroop test did not significantly differ between the three groups [main effect of group, *F*_(2, 93)_ = *0.594, p* = *0.554*, partial η^2^ = 0.013]. A significant main effect of time was observed, with faster response times at post-test (813 ± 19 ms) compared to pre-test [847 ± 18 ms; *F*_(1, 93)_ = *4.61, p* = *0.034*, partial η^2^ = 0.047]. The group × time interaction was also statistically significant [*F*_(2, 93)_ = *5.16, p* = *0.007*, partial η^2^ = 0.100], indicating that the pattern of change over time differed among the groups (Figure 3A). This interaction remained significant after FDR correction (*p*_FDR_ =0.025).

Bonferroni-adjusted simple effects analyses confirmed a significant decrease in response time from pre-test to post-test in the squat imagery group by 103.14 ms [*SE* = *25.61, p*<*0.001*, 95% CI (52.28, 154.00)], with no significant change in either the squat execution group (*p* = *0.953*) or the control group (*p* = *0.90*4). Comparisons of change scores further indicated that the pre-to-post reduction was significantly greater in the squat imagery group than in both the control group [mean difference = 106.54 ms, *p* = *0.018*, 95% CI (13.91, 199.16)] and the squat execution group [mean difference = 101.47 ms, *p* = *0.027*, 95% CI (8.85, 194.10)]. The execution and control groups did not differ in change scores (*p* = *1.000*).

A significant main effect of time was also found in the complex level of the Stroop task [*F*_(1, 93)_ = *21.74, p*<*0.001*, partial η^2^ = 0.189], with response times decreasing from pre-test (1,178 ± 27 ms) to post-test (1,087 ± 25 ms), though there was no main effect of group [*F*_(2, 93)_ = *0.861, p* = *0.426*]. However, the group × time interaction was significant [*F*_(2, 93)_ = *6.87, p* = *0.002*, partial η^2^ = 0.129], indicating a different change between groups over time. Upon further inspection, response times improved from pre-test to post-test in both the imagery and execution groups, but remained similar in the control group (Figure 3B). This interaction remained significant after FDR correction (*p*_FDR_ = 0.012).

Follow-up analyses indicated that response time decreased from pre-test to post-test in both the squat imagery group [mean difference = 164.72 ms, *SE* = *31.57, p*<*0.001*, 95% CI (102.03, 227.42)] and the squat execution group [mean difference = 113.07 ms, *SE* = *34.59, p* = *0.002*, 95% CI (44.39, 181.75)], but not in the control group (*p* = 0.855). The reduction was greater in the squat imagery group than in the control group [mean difference = 171.06 ms, *p* = *0.001*, 95% CI (56.88, 285.23)], whereas the difference between the squat execution and control groups was at the threshold of statistical significance [mean difference = 119.40 ms, *p* = *0.050*, 95% CI (0.15, 238.65)]. No significant difference in change was found between the squat imagery and squat execution groups (*p* = *0.819*).

#### 3.1.2 Accuracy

A significant main effect of time was found for accuracy in the simple level of the Stroop test [*F*_(1, 93)_ = *4.39, p* = *0.039*, partial η^2^ = 0.045], with accuracy declining slightly from pre-test (99.0 ± 0.3%) to post-test (97.7 ± 0.6%). There was no significant main effect of group [*F*_(2, 93)_ = *0.37, p* = 0.695], and no group × time interaction [*F*_(2, 93)_ = *1.12, p* = *0.332*].

Similarly, accuracy in the complex Stroop condition declined slightly from pre-test (97.6 ± 0.3%) to post-test (96.6 ± 0.5%), with a significant main effect of time [*F*_(1, 93)_ = *4.72, p* = *0.032*, partial η^2^ = 0.048]. However, there was no significant main effect of group [*F*_(2, 93)_ = *0.29, p* = *0.748*], nor a significant group × time interaction [*F*_(2, 93)_ = *0.09, p* = *0.913*].

### 3.2 Sternberg paradigm

#### 3.2.1 Response time

**Figure 4:**
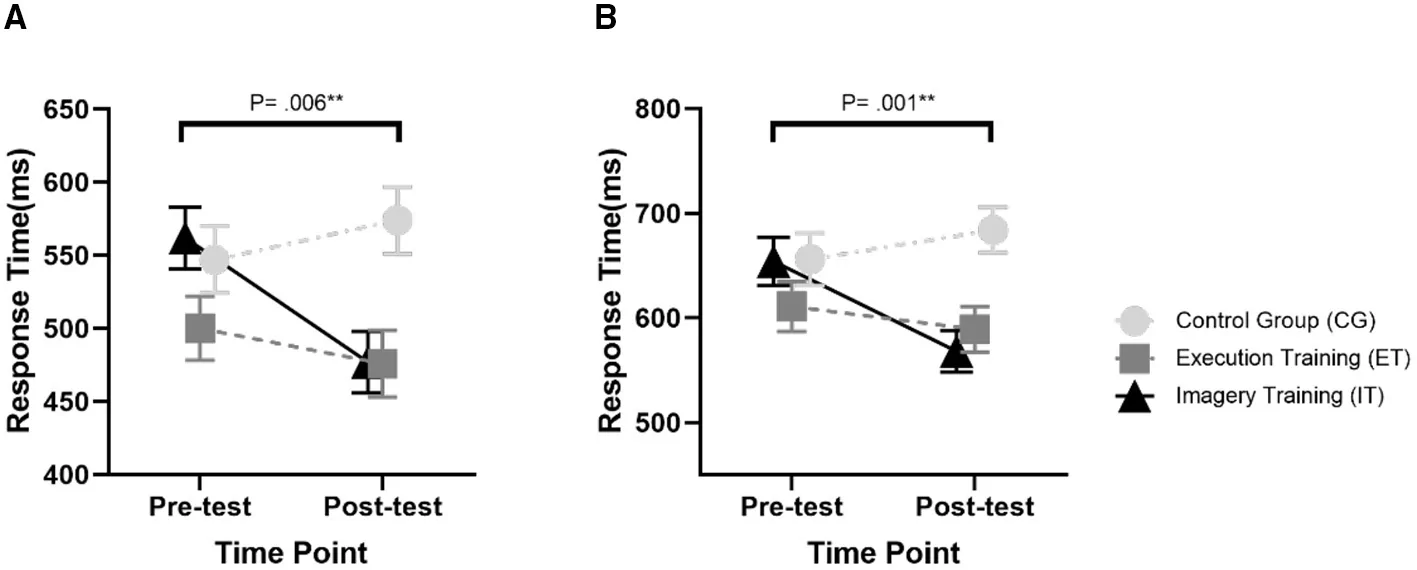
Response time (±SEM) for each group across pre- and post-test sessions in the Sternberg Paradigm task. **(A)** Shows the one-item condition, and **(B)** shows the three-item condition. IT, squat imagery group; ET, squat execution group; CG, control group. The *p*-values shown in the figure indicate the original group × time interaction effects; FDR-adjusted *p*-values are reported in the text. A significant group × time interaction was observed (*P* = .006; *P* = .001).

There was a significant main effect of time on response time in the one-item condition of the Sternberg task [*F*_(1, 95)_ = *3.96, p* = *0.049*, partial η^2^ = 0.040], with faster response at post-test (509 ± 13 ms) than pre-test (536 ± 13 ms). There was a significant main effect of group on response time in the one-item condition of the Sternberg task [*F*_(2, 95)_ = *3.64, p* = *0.030*, partial η^2^ = 0.071]. Descriptively, response times were slowest in the control group (560 ± 19 ms), faster in the squat imagery group (520 ± 18 ms), and fastest in the squat execution group (488 ± 19 ms). A significant group × time interaction was observed [*F*_(2, 95)_ = *5.49, p* = *0.006*, partial η^2^ = 0.104], indicating that the magnitude and direction of change in response time over time varied between groups (Figure 4A). This interaction remained significant after FDR correction (*p*_FDR_ = 0.025).

Follow-up analyses showed that response time decreased significantly from pre-test to post-test in the squat imagery group by 84.58 ms [*SE* = 22.72, *p* < 0.001, 95% CI (39.48, 129.69)], whereas no significant change was observed in the squat execution group (*p* = 0.314) or the control group (*p* = 0.290). Because the homogeneity of variance assumption was not met, Welch's ANOVA and Games–Howell comparisons were used for the change scores. The overall difference in change scores was significant, *F*_(2, 58.34)_ = 4.59, *p* = 0.014. The reduction in response time was greater in the squat imagery group than in the control group [mean difference = 111.08 ms, *p* = 0.017, 95% CI (16.99, 205.18)], but did not differ significantly from the squat execution group (*p* = 0.074). No significant difference was observed between the squat execution and control groups (*p* = 0.349). In the three-item condition of the Sternberg task, a significant main effect of time was observed [*F*_(1, 95)_ = *5.20, p* = *0.025*, partial η^2^ = 0.05], with faster response at post-test (613 ± 12 ms) than pre-test (640 ± 14 ms). Additionally, a significant main effect of group was also found [*F*_(2, 95)_ = *3.21, p* = *0.045*, partial η^2^ = 0.063]. Descriptively, mean response time was lowest in the squat execution group, followed by the squat imagery and control groups. A significant group × time interaction was found [*F*_(2, 95)_ = *7.96, p* = *0.001*, partial η^2^ = 0.14; Figure 4B], indicating that changes in response time over time differed between groups. This interaction remained significant after FDR correction (*p*_FDR_ = *0.009*).

Follow-up analyses showed that response time decreased significantly from pre-test to post-test in the squat imagery group by 86.42 ms [*SE* = 19.43, *p* < 0.001, 95% CI (47.85, 124.98)], whereas no significant change was observed in the squat execution group (*p* = 0.290) or the control group (*p* = 0.198). Because the homogeneity of variance assumption was not met, Welch's ANOVA and Games–Howell comparisons were used for the change scores. The overall difference in change scores was significant, *F*_(2, 58.44)_ = 7.25, *p* = 0.002. The reduction in response time was greater in the squat imagery group than in both the control group [mean difference = 114.02 ms, *p* = 0.004, 95% CI (33.06, 194.97)] and the squat execution group [mean difference = 64.48 ms, *p* = 0.013, 95% CI (11.40, 117.56)]. No significant difference in change scores was found between the squat execution and control groups (*p* = 0.287).

Overall, response times on the five-item level of the Sternberg paradigm improved from pre-test to post-test [*F*_(1, 95)_ = *9.08, p* = *0.003*, partial η^2^ = 0.087], with faster response at post-test (729 ± 17 ms) than pre-test (782 ± 21 ms). However, a significant main effect of group was not found [*F*_(2, 95)_ = *0.85, p* = *0.432*], nor was there a group × time interaction [*F*_(2, 95)_ = *2.66, p* = *0.075*].

#### 3.2.2 Accuracy

There was no effect of group, time, or group^*^time interaction for accuracy on the one-item condition of the Sternberg paradigm [main effect of group, *F*_(2, 95)_ = *1.07, p* = *0.349*; main effect of time, *F*_(1, 95)_ = *0.14, p* = *0.710*; group × time interaction, *F*_(2, 95)_ = *1.44, p*= *0.242*].

On the three-item level of the Sternberg paradigm, a significant main effect of group was observed [*F*_(2, 95)_ = *4.05, p* = *0.021*, partial η^2^ = 0.079]. Descriptively, overall accuracy was higher in the squat imagery (98.3 ± 0.5%) and squat execution (98.3 ± 0.6%) groups than in the control group (96.3 ± 0.6%). No significant main effect of time [*F*_(1, 95)_ = *1.07, p* = 0.304] or group × time interaction was detected [*F*_(2, 95)_ = *0.95, p* = *0.390*].

For the five-item condition, a significant main effect of group was found, [*F*_(2, 95)_ = *3.39, p* = *0.038*, partial η^2^ = 0.067], with the squat imagery group (96.8 ± 0.8%) showing highest accuracy, followed by the squat execution group (96.3 ± 0.9%) and the control group (93.8 ± 0.9%). However, no significant main effect of time [*F*_(1, 95)_ = *0.77, p* = *0.382*], or group × time interaction [*F*_(2, 95)_ = *1.22, p* = *0.301*], was observed.

### 3.3 Flanker test

#### 3.3.1 Response time

Response times on the congruent stimuli of the Flanker task significantly improved from pre-test to the post-test [main effect of time, *F*_(1, 95)_ = *18.79, p*<*0.001*, partial η^2^ = 0.165; pre-test: 561 ± 10 ms, post-test: 524 ± 10 ms]. Meanwhile, a main effect of group was also observed [*F*_(2, 95)_ = *8.19, p* = *0.001*, partial η^2^ = 0.147), with both the imagery group (512 ± 14 ms) and the execution group (523 ± 15 ms) responded significantly faster than the control group (592 ± 16 ms; *p* = *0.002, 0.005, respectively*). However, the pattern of change in response time over time did not significantly differ between groups [group × time interaction, *F*_(2, 95)_ =*1.44, p* = *0.243*].

Overall, response times on the incongruent stimuli of the Flanker task also showed significant improvement across time [main effect of time, *F*_(1, 92)_ = *34.28, p*<*0.001*, partial η^2^ = 0.271], with post-test responses (616 ± 18 ms) faster than pre-test responses (697 ± 24 ms). There was a significant main effect of group [*F*_(2, 92)_ = 10.07, *p* < 0.001, partial η^2^ = 0.180]. Descriptively, response times were lowest in the squat execution group (pre-test: 607 ± 43 ms; post-test: 560 ± 33 ms), followed by the squat imagery group (pre-test: 638 ± 39 ms; post-test: 557 ± 30 ms) and the control group (pre-test: 847 ± 44 ms; post-test: 733 ± 33 ms). However, the pattern of change in response time over time did not significantly differ between groups [group × time interaction, *F*_(2, 92)_ = *1.88, p* = *0.165*].

#### 3.3.2 Accuracy

Accuracy on the congruent stimuli of the Flanker task revealed no significant main effect of group [*F*_(2, 95)_ = *0.43, p* = *0.651*], and no main effect of time [*F*_(1, 95)_ = *0.01, p* = *0.942*]. There was also no significant group × time interaction [*F*_(2, 95)_ = *0.83, p* = *0.438*].

On the incongruent condition, accuracy was similar over time [main effect of time, *F*_(1, 95)_ = *1.09, p* = *0.300*], and between groups [main effect of group, *F*_(2, 95)_ = *0.783, p* = *0.460*]. A group × time interaction was initially observed for incongruent accuracy, *F*_(2, 95)_ = *3.56, p* = *0.032*, partial η^2^ = 0.070. However, this interaction did not remain significant after FDR correction (*p*_FDR_ = *0.082*). Therefore, the descriptive differences between groups were not interpreted as robust intervention-related effects.

### 3.4 Visual Search test

#### 3.4.1 Response time

**Figure 5:**
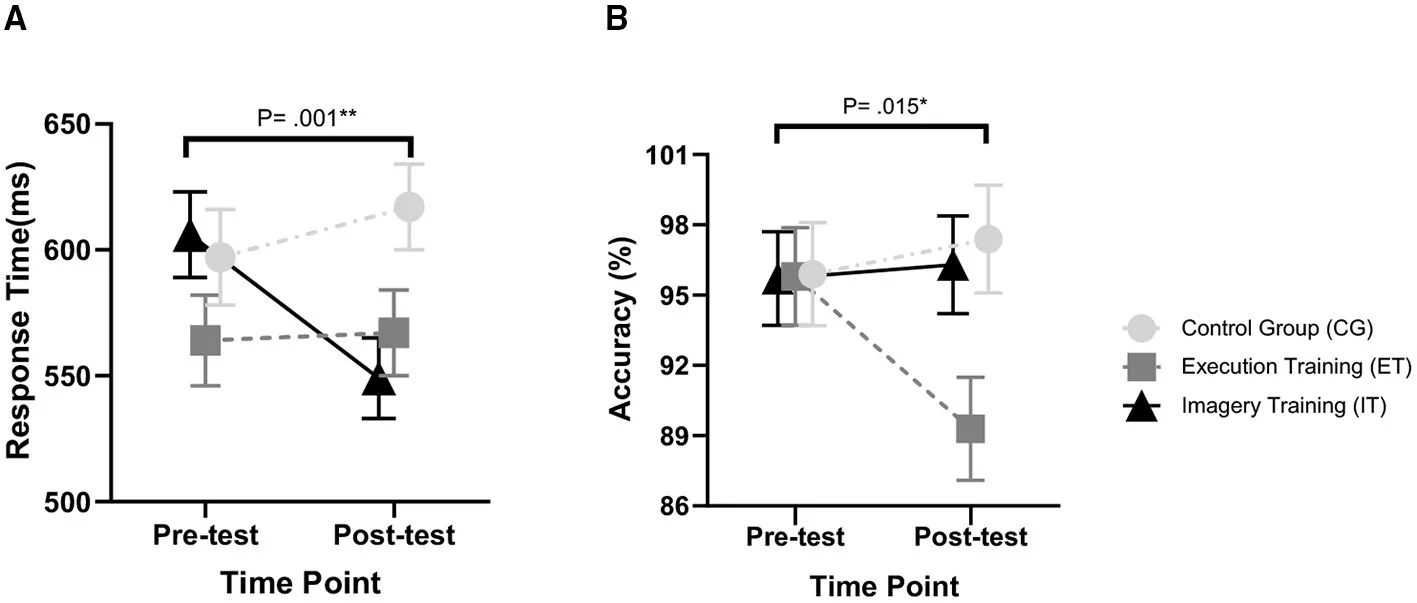
Performance changes across pre- and post-test sessions in the Visual Search task. **(A)** Shows response time in the congruent condition, and **(B)** shows accuracy in the incongruent condition. IT, squat imagery group; ET, squat execution group; CG, control group. The *p*-values shown in the figure indicate the original group × time interaction effects; FDR-adjusted *p*-values are reported in the text. A significant group × time interaction was observed (*P* = .001; *P* = .015).

Response time on the congruent condition of the Visual Search test revealed no significant main effect of time [*F*_(1, 95)_ = *1.72, p* = *0.192*], and no main effect of group [*F*_(2, 95)_ = *1.83, p* = *0.167*]. However, a significant group × time interaction was observed [*F*_(2, 95)_ = *7.43, p* = *0.001*, partial η^2^ = 0.135; Figure 5A]. Further inspection revealed that response times decreased from pre- to post-test in the squat imagery group, remained similar over time in the squat execution group, and slowed slightly in the control group. This interaction remained significant after FDR correction (*p*_FDR_ = *0.009*).

Follow-up analyses showed that response time decreased from pre-test to post-test in the squat imagery group by 57.25 ms [*SE* = 14.40, *p* < 0.001, 95% CI (28.67, 85.83)], whereas no significant change was observed in the squat execution group (*p* = 0.851) or the control group (*p* = 0.210). The reduction was greater in the squat imagery group than in both the control group [mean difference = 77.15 ms, *p* = 0.001, 95% CI (25.10, 129.20)] and the squat execution group [mean difference = 60.13 ms, *p* = 0.015, 95% CI (8.97, 111.28)]. No significant difference in change scores was found between the squat execution and control groups (*p* = 1.000).

For the incongruent condition, a significant main effect of time was found [*F*_(1, 95)_ = *4.56, p* = *0.035*, partial η^2^ = 0.046], with post-test responses (1,148 ± 29 ms) faster than pre-test responses (1,194 ± 31 ms). A significant main effect of group was also observed [*F*_(2, 95)_ = *6.62, p* = *0.002*, partial η^2^ = 0.122], with mean response times of 1,311 ± 50 ms for the control group, 1,142 ± 46 ms for the imagery group, and 1,060 ± 49 ms for the execution group. However, the group × time interaction was not significant [*F*_(2, 95)_ = *1.10, p* = *0.337*].

#### 3.4.2 Accuracy

Accuracy on the congruent condition of the Visual Search test revealed no significant main effect of group [*F*_(2, 95)_ = *0.20, p* = *0.819*], and no main effect of time [*F*_(1, 95)_ = *1.19, p* = *0.278*]. Furthermore, no significant group × time interaction was observed [*F*_(2, 95)_ = *1.65, p* = *0.198*].

Overall, accuracy on the incongruent condition of the Visual Search task was not affected by group [main effect of group, *F*_(2, 95)_ = *1.32, p* = *0.272*] or time [main effect of time, *F*_(1, 95)_ = *1.51, p* = *0.222*]. In contrast, a significant group × time interaction was observed [*F*_(2, 95)_ = *4.378, p* = *0.015*, partial η^2^ = 0.084]. Further inspection revealed that accuracy increased slightly over time in the squat imagery (from 95.7 ± 2.0% to 96.3 ± 2.1%) and the control group (from 95.8 ± 2.2% to 97.3 ± 2.3%), whereas accuracy decreased slightly over time in the squat execution group (Figure 5B). This interaction remained significant after FDR correction (*p*_FDR_ = *0.045*).

Follow-up analyses showed that accuracy decreased significantly from pre-test to post-test in the squat execution group by 6.44 percentage points [*SE* = 2.10, *p* = 0.002, 95% CI (2.30, 10.60)], whereas no significant change was observed in the squat imagery group (*p* = 0.782) or the control group (*p* = 0.481). Bonferroni-adjusted comparisons of change scores indicated that the decline in accuracy was greater in the squat execution group than in both the control group [mean difference = 7.96 percentage points, *p* =0.027, 95% CI (0.69, 15.22)] and the squat imagery group [mean difference = 6.99 percentage points, *p* = 0.048, 95% CI (0.04, 13.93)]. No significant difference in change was found between the squat imagery and control groups (*p* = 1.000).

## 4 Discussion

This study examined the acute effects of squat imagery and squat execution on cognitive function in collegiate athletes. The findings showed that squat imagery was associated with significant improvements in selected measures of response time across inhibitory control, working memory, and visual attention. Squat execution also produced improvements in some outcomes, although the pattern was less consistent. Direct comparisons indicated that the squat imagery group showed greater improvements than the squat execution group only for selected response-time outcomes. Therefore, the findings support a potential acute cognitive benefit of squat imagery, but do not demonstrate its general superiority over squat execution.

In the Stroop task, the squat imagery group showed significant reductions in response time under both the simple and complex conditions. The squat execution group also showed a significant reduction in the complex condition, whereas no significant response-time change was observed in the control group. Direct comparisons of change scores showed a greater reduction in the squat imagery group than in the squat execution group for the simple condition, but not for the complex condition. Accuracy declined slightly but significantly over time in both Stroop conditions, although the absence of significant group × time interactions indicated that this decline was not specific to the squat imagery condition. Therefore, the faster response times should be interpreted cautiously, as a possible speed–accuracy trade-off or repeated-testing effect cannot be excluded. Previous research has similarly reported associations between imagery-based interventions and Stroop performance (Zemla et al., 2023), although the present findings should be interpreted as behavioral evidence rather than direct evidence of improved neural efficiency. Overall, the results suggest that acute squat imagery may support selected aspects of processing speed and inhibitory control, but the findings do not establish a consistent advantage over squat execution.

Another key finding of the present study was that squat imagery was associated with improved response time in the Sternberg working memory task. Significant group × time interactions were observed in both the one-item and three-item conditions. In the one-item condition, response time decreased significantly in the squat imagery group, whereas no significant change was observed in the squat execution or control groups. However, the reduction in the squat imagery group was significantly greater than that in the control group, but not significantly greater than that in the squat execution group. In the three-item condition, the squat imagery group showed a significant reduction in response time, and this improvement was significantly greater than the changes observed in both the squat execution and control groups. These findings suggest that acute squat imagery may support working memory performance, particularly under the three-item condition. Previous research has also reported that both motor imagery and physical exercise may influence working memory performance (Anders et al., 2021; Haire and Hagan, 2021). Nevertheless, the present findings indicate a specific advantage of squat imagery over physical execution only for the three-item condition, rather than a consistent superiority across all working memory outcomes.

In the Flanker task, response times decreased over time in both the congruent and incongruent conditions; however, the absence of significant group × time interactions indicates that these changes were not specific to either the squat imagery or squat execution condition. Similarly, although the group × time interaction for incongruent accuracy was significant before correction, it did not remain significant after FDR correction. Therefore, the observed descriptive differences in accuracy should not be interpreted as robust intervention-related effects. Taken together, the Flanker findings did not provide robust evidence that either acute squat imagery or squat execution produced a specific benefit in attentional control under interference. The overall reduction in response time may instead reflect repeated task exposure or general practice effects.

In the Visual Search task, the squat imagery group showed a significant reduction in response time in the congruent condition, and this improvement was significantly greater than the changes observed in both the squat execution and control groups. No corresponding group × time interaction was found for response time in the incongruent condition, indicating that the benefit was observed only in the congruent condition rather than across both task conditions. Accuracy in the congruent condition did not differ significantly over time among the groups. In the incongruent condition, the significant group × time interaction was driven by a decline in accuracy in the squat execution group, whereas accuracy remained stable in the squat imagery and control groups. Therefore, this result should not be interpreted as an improvement in accuracy following squat imagery, but rather as evidence that performance was maintained in the imagery condition while declining after physical execution. Overall, the findings suggest that acute squat imagery may selectively facilitate visual response speed under lower attentional demand, although its benefits did not extend consistently to the more complex incongruent condition. This interpretation is consistent with evidence that imagery and attention interact during perceptual processing (Kosslyn et al., 1999; Moriya, 2018), but the present behavioral results do not permit conclusions regarding specific neural mechanisms.

Across tasks, the selective behavioral improvements observed following squat imagery may be interpreted within the broader framework of frontoparietal control systems that support goal-directed cognition (Cole et al., 2014). Working memory, attentional allocation, and inhibitory control are thought to depend on coordinated activity across frontal and parietal regions involved in maintaining internal representations, monitoring conflict, and allocating cognitive resources according to task demands (Fuster, 2009; Rushworth et al., 2004). Previous neuroimaging research has shown that motor imagery and physical execution recruit partially overlapping frontal and parietal regions associated with attentional control, response selection, and inhibition (Aron et al., 2003; Yanagisawa et al., 2010). Because motor imagery requires sustained attention to internally generated movement representations, engagement of these shared cognitive-control processes may provide one possible explanation for the improvements observed in selected response-time outcomes. However, no neuroimaging, electrophysiological, or physiological measures were collected in the present study. Therefore, the involvement of specific brain regions or neural networks cannot be inferred directly from the current behavioral findings and should be regarded as a theoretical possibility requiring confirmation in future studies using methods such as functional Magnetic Resonance Imaging (fMRI), Electroencephalography (EEG), or Functional near-infrared spectroscopy (fNIRS).

Although the present protocol involved motor imagery alone rather than combined action observation and motor imagery, the broader Action Observation and Motor Imagery literature provides relevant context for understanding the use of motor simulation in sport. Previous Action Observation and Motor Imagery (AOMI) research suggests that observed and internally simulated actions may support motor representations and performance-related processes (Çiftçi and Ylmaz, 2024). In the present study, the squat was selected because it is a familiar and fundamental lower-body movement that can be represented consistently by athletes from different sporting backgrounds. Its relative simplicity and familiarity may have facilitated the generation of stable movement representations without requiring overt physical execution.

Overall, this study extends existing research by examining the acute effects of squat imagery and squat execution across multiple cognitive domains in collegiate athletes. The present findings highlight that a single session of squat imagery may produce short-term benefits in selected measures of inhibitory control, working memory, and visual attention. However, these effects were not consistent across all tasks and outcomes, and squat imagery was not uniformly superior to squat execution. From an applied perspective, squat imagery may represent a low-physical-load strategy that could be considered when additional physical exertion is undesirable, such as during periods of intensive training or injury rehabilitation. The observed reductions in response time represent measurable improvements under laboratory conditions and may be relevant to sporting situations requiring rapid information processing and response selection, although direct transfer to sport-specific performance was not assessed in the present study. Nevertheless, their practical significance and transfer to real-world sporting performance remain to be established. Future studies should incorporate sport-specific cognitive tasks and performance measures to evaluate the ecological validity of these effects.

Several limitations should be considered when interpreting the findings. First, the study examined the immediate effects of a single intervention session; therefore, the results primarily reflect short-term changes and cannot determine whether similar effects would persist following longer-term practice. In addition, the same cognitive task paradigms and fixed task order were used at pre-test and post-test. This approach helped ensure that any potential order or fatigue effects were consistent across repeated administrations and experimental groups; however, it did not eliminate the possibility that practice, order, or fatigue effects influenced performance. Although the specific stimuli were randomly generated or randomly presented at each administration, future studies could further address this issue by using fully parallel task versions, counterbalanced task orders, and longer follow-up periods. Although a familiarization session and standardized administration procedures were used to reduce procedural variability, some practice, order, or fatigue effects may still have influenced performance. Future studies could address these issues by using alternate task versions, counterbalanced task order, and longer follow-up periods.

A further limitation concerns the assessment of individual differences in imagery and intervention engagement. Although imagery-related characteristics were assessed as part of the broader research project, these data could not be linked reliably to randomized group allocation, and no immediate manipulation check was administered after the imagery session. Expectancy and arousal were not assessed directly, and their potential contribution to the observed behavioral changes therefore cannot be excluded. Consequently, the possible influence of imagery ability, vividness, and engagement could not be examined directly. Moreover, the study relied on behavioral outcomes, and the neural explanations discussed should therefore be regarded as theoretical interpretations rather than mechanisms demonstrated by the present data. Finally, demographic and training-related characteristics were available for the overall sample but could not be compared across the three randomized groups because of the data-linkage procedure. Although random allocation reduced the likelihood of systematic group differences, baseline comparability could not be confirmed directly.

## 5 Conclusion

This study demonstrates that acute squat imagery and squat execution may influence selected aspects of cognitive performance in collegiate athletes, with squat imagery producing greater improvements than squat execution in some response-time outcomes. The findings indicate that squat imagery may offer a practical, low-physical-load strategy for supporting inhibitory control, working memory, and visual attention when additional physical exertion is undesirable. However, the effects were not consistent across all cognitive outcomes, and squat imagery was not uniformly superior to squat execution. Overall, the study contributes to the growing evidence that motor imagery may have value as an adjunct to athletic training, while further research is needed to determine the persistence and practical transfer of these acute effects.

## Data Availability

The original contributions presented in the study are included in the article/supplementary material, further inquiries can be directed to the corresponding author.
